# Degradation of chlorophyll and synthesis of flavonols during autumn senescence—the story told by individual leaves

**DOI:** 10.1093/aobpla/ply028

**Published:** 2018-05-04

**Authors:** Heta Mattila, Dimitar Valev, Vesa Havurinne, Sergey Khorobrykh, Olli Virtanen, Mikko Antinluoma, Kumud B Mishra, Esa Tyystjärvi

**Affiliations:** 1 Department of Biochemistry/Molecular Plant Biology, University of Turku, Turku, Finland; 2 Global Change Research Institute, CAS, Bělidla, Brno, Czech Republic

**Keywords:** Bird cherry, chlorophyll breakdown, circadian rhythm, diurnal, maple, rowan, silver birch

## Abstract

Autumn senescence of deciduous trees is characterized by chlorophyll degradation and flavonoid synthesis. In the present study, chlorophyll and flavonol contents were measured every morning and evening during the whole autumn with a non-destructive method from individual leaves of *Sorbus aucuparia*, *Acer platanoides*, *Betula pendula* and *Prunus padus*. In most of the studied trees, the chlorophyll content of each individual leaf remained constant until a phase of rapid degradation commenced. The fast phase lasted only ~1 week and ended with abscission. In *S. aucuparia*, contrary to the other species, the chlorophyll content of leaflets slowly but steadily decreased during the whole autumn, but rapid chlorophyll degradation commenced only prior to leaflet abscission also in this species. An increase in flavonols commonly accompanied the rapid degradation of chlorophyll. The results may suggest that each individual tree leaf retains its photosynthetic activity, reflected by a high chlorophyll content, until a rapid phase of chlorophyll degradation and flavonoid synthesis begins. Therefore, in studies of autumn senescence, leaves whose chlorophyll content is decreasing and leaves with summertime chlorophyll content (i.e. the leaves that have not yet started to degrade chlorophyll) should be treated separately.

## Teaser

Chlorophylls are degraded and flavonoids synthesized during autumn senescence of deciduous trees. In the present study, chlorophyll and flavonol contents of individual leaves were monitored non-destructively throughout the autumn. Loss of chlorophyll and synthesis of flavonols were not gradual. Instead, each leaf maintained steady chlorophyll content until rapid chlorophyll degradation, accompanied by flavonol synthesis, was triggered. In ~1 week, the leaf turns yellow and falls. The pattern was similar in birch (*Betula pendula*), maple (*Acer platanoides*) and bird cherry (*Prunus padus*); in rowan (*Sorbus aucuparia*), very slow gradual chlorophyll degradation occurred on top of the main pattern.

## Introduction

During autumn senescence, deciduous trees shed their leaves, remobilizing nutrients, mainly nitrogen and phosphorus (for review, see Brant and Chen 2015), for winter storage. Senescence is a genetically controlled, energy-consuming process that ultimately leads to death of specific plant organs (for review, see e.g. Hopkins *et al.* 2007).

The course of autumn senescence in *Populus tremula* has been described by Keskitalo *et al.* (2005). The first visible sign is chlorophyll (Chl) degradation, followed by degradation of other macromolecules (e.g. carotenoids and proteins), nutrient remobilization and cessation of starch accumulation. Photosynthesis declines while functional chloroplasts turn into gerontoplasts, and mitochondria become the main energy producers (e.g. Collier and Thibodeau 1995; Andersson *et al.* 2004).However, a small number of chloroplasts remain intact and some photosystem II (PSII) centres stay functional until almost the end of senescence (Keskitalo *et al.* 2005; Moy *et al.* 2015). Ultimately, metabolic activity ceases, cytoplasm is degraded, the abscission zone forms and the leaf falls off.

In *P. tremula*, autumn senescence seems to be triggered only by the shortening of the photoperiod (Fracheboud *et al.* 2009), but in other species both the onset and duration of senescence are affected by additional factors, such as temperature (Fu *et al.* 2014; Gill *et al.* 2015). During the growth period, senescence of a whole plant or a plant organ can be triggered by different stresses, darkness or developmental stage. The cellular processes at the early phases of senescence have been shown to differ remarkably depending on the triggering factor but at later stages the regulation seems to be similar (for review, see e.g. Fischer 2012; Guo and Gan 2012; Springer *et al.* 2015). During autumn senescence, expression levels of many genes change (Bhalerao *et al.* 2003; Andersson *et al.* 2004; Wen *et al.* 2015). Generally, genes related to photosynthesis are down-regulated, but many stress-related genes, as well as genes involved in degradation processes or in mitochondrial functions, are up-regulated or stay constant.

Up to 75 % of leaf nitrogen is in chloroplasts (Peoples and Dalling 1988), both in thylakoid proteins and in stromal proteins like rubisco. A significant part of the nitrogen is found in chlorophyll-binding light-harvesting complexes (LHC), and degradation of chlorophyll prevents the production of singlet oxygen, a reactive oxygen species (ROS), by free chlorophylls released when the protein is degraded. Breakdown of chlorophyll begins with reduction of chlorophyll *b* to chlorophyll *a* and proceeds via multiple steps to a colourless linear tetrapyrrole (for review, see Hörtensteiner and Kräutler 2011). Fluorescent chlorophyll catabolites are able to produce singlet oxygen (e.g. Pružinská *et al.* 2007) and are moved to the vacuole where they lose their colour in an acid-catalyzed reaction (Hörtensteiner and Kräutler 2011). Derivatives of the non-fluorescent degradation products and side products of the degradation have been speculated to play roles as antioxidants and signal molecules (Christ and Hörtensteiner 2014).

The spectacular autumn colours are partly caused by exposure of carotenoids due to faster degradation of chlorophyll. In addition, some species also synthesize specific flavonoids, e.g. anthocyanins (for review, see Falcone Ferreyra *et al.* 2012) which are largely responsible for the red colours in senescing leaves. Anthocyanins are suggested to act as antioxidants or metal ion chelators, in blocking ultraviolet (UV) or visible radiation, or playing roles in defence and signalling (for review, see Archetti 2009a; Landi *et al.* 2015). The amounts of other flavonoid compounds, flavonols or flavonol glycosides, have been shown to increase during age-related senescence (Torras-Claveria *et al.* 2012) but their roles are poorly understood. In contrast to anthocyanins, flavonols do not absorb visible light. They are found in the cuticle and sometimes inside the cells and they block UV radiation (for review, see Solovchenko and Merzlyak 2008) and may act as ROS-scavengers or signal molecules (Pollastri and Tattini 2011). Flavonol glycosides have also been suggested to be involved in super-cooling of xylem parenchyma cells of *Cercidiphyllum japonicum* (Kasuga *et al.* 2008). The diversity of plant flavonoids may suggest that different compounds have different roles.

Even though autumn senescence is considered to be coordinated and synchronized at the organism level, neither all leaves of a tree nor all cells of a single leaf senesce simultaneously (e.g. Keskitalo *et al.* 2005). However, in many studies, autumn senescence is treated as an averaged phenomenon, and investigations that connect senescence of an individual leaf and the whole tree are rare. In the present study, we followed a number of single leaves of four trees, each representing a different species, *Sorbus aucuparia*, *Acer platanoides*, *Betula pendula* and *Prunus padus*, from late growth season through the whole autumn. The chosen trees are native to Northern Europe and all of them except *B. pendula* turn red during senescence (Archetti 2009b), probably due to the accumulation of anthocyanins. The aims of the study were to find out how the chlorophyll content of an individual leaf behaves during autumn senescence both on a day-to-day basis and during the circadian cycle, and to study the role of flavonols in tree senescence.

## Methods

### Plant material

Four deciduous trees, rowan (*S. aucuparia*; ~5 m tall), Norway maple (*A. platanoides*; ~10 m), silver birch (*B. pendula*; ~15 m) and bird cherry (*P. padus*; ~1 m), growing in a small park (Turku, Finland, 60°N, 22°E), were chosen for the measurements, and individual full-grown leaves from each tree were marked before the measurements started. The park is located on the top of a small hill, and the environment is sunny and dry. The park was not directly illuminated by street lights. Before the measurements started, several leaves from each tree (at 0.5‒2 m height) were marked. *Acer platanoides* and *B. pendula* trees were not shaded by other trees much; though the lower leaves of *A. platanoides* marked in the present study were shaded by the upper leaves of the same tree. *Sorbus aucuparia* and *P. padus* trees were partially shaded by the other trees in the area, depending on the time of day and the date.

Weather data **[see Supporting Information—Fig. S1]** were obtained from Finnish Meteorological Institute; the weather station is located 7 km from the studied trees.

### Pigment measurements

#### Optical measurements.

Chlorophyll and epidermal flavonol contents were measured simultaneously *in vivo* using a non-destructive measurement device (Dualex Scientific™, Force-A, Paris, France). The indexes of chlorophyll and flavonols are defined as (http://www.force-a.com/en, 11 November 2016; for the exact formula for chlorophylls, see Cerovic *et al.* 2012):

$$Chlorophyll=\frac{\text{transmitted near infrared light }-\text{transmitted red light}}{\text{transmitted red light}}$$

$$Flavonols=\text{Log}\frac{\text{near infrared fluorescence excited by red light}}{\text{near infrared fluorescence excited by UV-A}}$$

The measurements were taken from four leaves consisting of 57 leaflets (1 measurement per leaflet) of *S. aucuparia*, 15 leaves (3 measurements per leaf) of *A. platanoides*, 30 leaves belonging to six branches (2 measurements per leaf) of *B. pendula* and 11 leaves (6 measurements per leaf) of *P. padus*. The measurements were conducted daily (excluding weekends) at sunrise and at sunset, from 24 August 2015 to 22 October 2015 or until the leaf or leaflet fell off or was damaged by an external factor like herbivory.

#### Isolation of pigments.

The chlorophyll contents of *S. aucuparia* leaflets were measured also with a destructive method during 23 August‒25 September 2009. Ten leaf discs were collected twice a day, at sunrise and at sunset, in a predetermined random order from 125 previously marked leaves. Chlorophylls were extracted by incubating the leaf discs in *N*,*N*-dimethylformamide (DMF) for 2‒3 days in darkness at 4 °C, after which the amount of chlorophyll was quantified spectrophotometrically according to Porra *et al.* (1989). In the measurements done in 2015, chlorophylls of several leaves from all four trees with varying greenness were measured both with Dualex and extracted in DMF to validate and calibrate the non-destructive method for senescent leaves.

### High-performance liquid chromatography analysis of phenolic compounds

Leaves were dried at room temperature for 24 h in a desiccator and stored at 4 °C. Large veins were removed and the rest of the leaf material (dry weight 5‒50 mg) was ground to small pieces, 1 mL of methanol was added, the sample was vortexed and incubated over night at 4 °C in darkness. Thereafter, the sample was vortexed again, centrifuged, and the supernatant was removed. Another 1 mL of methanol was added, the sample was vortexed and centrifuged and the supernatant was combined with the previous extract. The sample was filtered through a 0.2-µm polytetrafluoroethylene filter and assayed with high-performance liquid chromatography (HPLC; Agilent 1100 Series, Agilent Technologies, Germany) according to Seal (2016), except that a LiChroCART column (RP-18, 5 µm particle size; 125 × 4 mm) was used, and the gradient elution time was increased from 55 to 70 min to remove carotenoids from the column. Estimates of the total amounts of flavonols and other phenolic compounds were calculated by summing the absorbance at 355 nm of each peak **(see Supporting Information—Fig. S2** for the retention times and the spectra), and normalized by dividing by the dry weight of the sample. Due to different grinding methods, the amount of phenolics cannot be compared between the species. The compounds were manually classified as a flavonol, flavanone, other flavonoid, phenolic acid or other related compound according to their retention times and absorption spectra **[see Supporting Information—Fig. S2]**.

### Statistical analyses

Mann–Whitney *U* test and Student’s *t*-test were performed in Excel (Microsoft). Two-tailed distributions were used, and significant differences are reported only when the null hypothesis was rejected with probability of 99 % or higher. The sample sizes are given in respective figures. Analysis of variance (ANOVA) was calculated with R software (R Core Team 2014).

For the analysis of the circadian variation in the change of chlorophyll and flavonoid contents of the leaves, each measurement from all four trees was manually classified to separate samples in which rapid chlorophyll degradation had started or to samples in which rapid chlorophyll degradation had not started (stable group). In *S. aucuparia*, leaves 1 and 2, three leaflets of leaf 3 and two leaflets of leaf 4 were classified to the rapid group from the beginning of the analysis. All other leaflets of leaf 3 belonged to the rapid group from 15 September on, and all other leaflets of leaf 4 belonged to the rapid group from 21 September on. In *A. platanoides*, all leaves belonged to the rapid group from 12 September on. In *B. pendula*, two leaves of the first leaf group were classified as rapid from 28 August on, two leaves from 25 September on and two from 16 October on. In *P. padus*, all leaves of the second analysis group were classified as rapid. All other measurements belonged to the stable group.

## Results

### Chlorophyll contents of individual leaves of senescing trees remained stable until onset of rapid degradation

**Figure 1.**
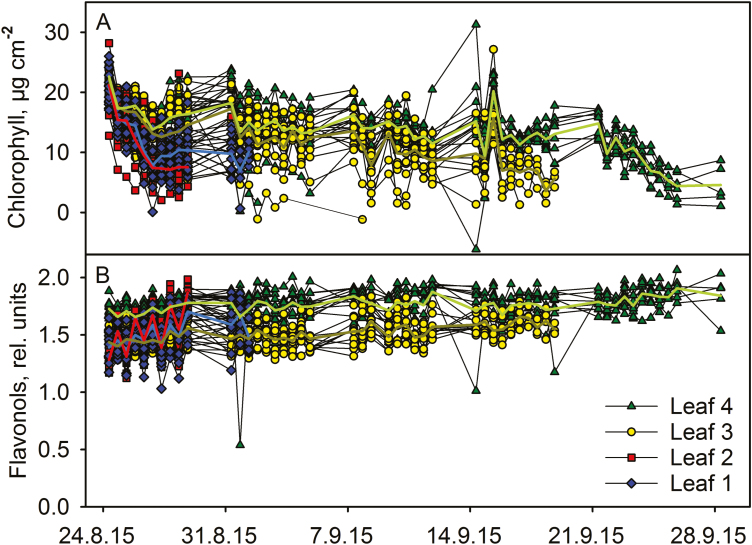
Chlorophyll (A) and flavonol (B) contents during autumn senescence in 57 *Sorbus aucuparia* leaflets belonging to four leaves. The numbering of the leaves indicates their respective order in the tree, the smallest being lowest. The symbols with the same colour in (A) and (B) represent the same leaf, and each line shows an average of the leaflets of one leaf.

**Figure 2.**
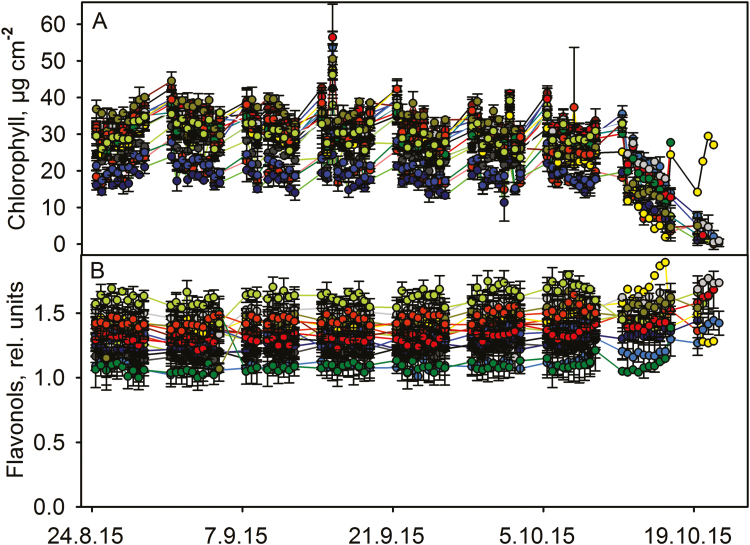
Chlorophyll (A) and flavonol (B) contents during autumn senescence in 15 *Acer platanoides* leaves, shown with different colours. The curves with the same colour in (A) and (B) represent the same leaf. Error bars show SD calculated from three measurements from the same leaf.

**Figure 3.**
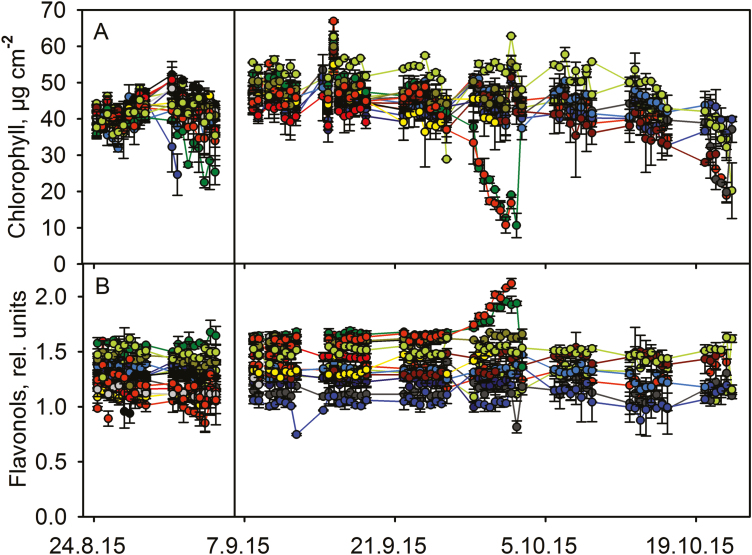
Chlorophyll (A) and flavonol (B) contents during autumn senescence in 15 (first set) and 14 (second set) *Betula pendula* leaves, shown with different colours. Measurements before and after the vertical line are from different leaves; the first set of leaves was abandoned because of extensive damage by insect herbivores. The curves with the same colour in (A) and (B) represent the same leaf. Error bars show SD calculated from two measurements from the same leaf.

**Figure 4.**
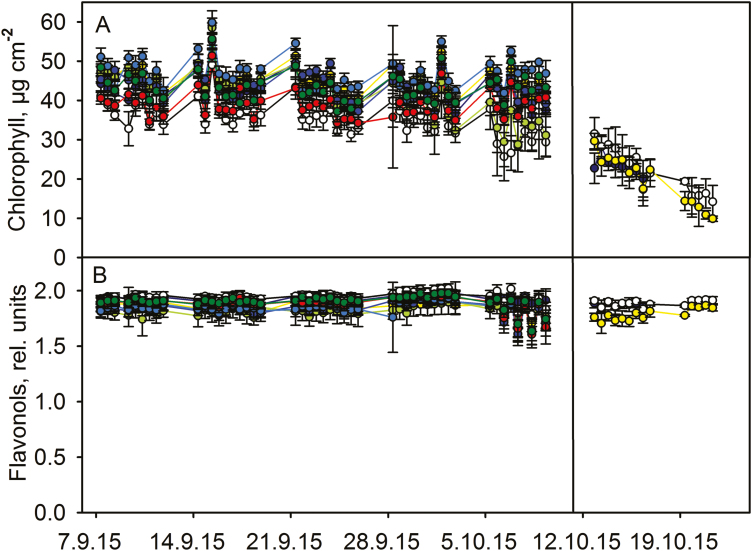
Chlorophyll (A) and flavonol (B) contents during autumn senescence in eight (first set) and three (second set) *Prunus padus* leaves, shown with different colours. Measurements before and after the vertical line are from different leaves; the first set of leaves was abandoned because of massive damage to the leaf marks by an herbivore or a passer-by. The curves with the same colour in (A) and (B) represent the same leaf. The error bars show SD calculated from six measurements from the same leaf.

Chlorophyll and flavonol contents of individual leaves of *S. aucuparia*, *A. platanoides*, *B. pendula* and *P. padus* were recorded with a non-destructive method twice a day from 23 August 2015 to 23 October 2015 (Figs 1‒4). The autumn weather was typical for the region, with day temperatures falling from around 15 to 10 °C during the experiment, and the first night frosts in mid-October **[see Supporting Information—Fig. S1]**.

The optical method was validated for senescing leaves and was found to work well (see Appendix 1). In addition, the chlorophylls of leaflets of *S. aucuparia* were analysed with a destructive method during autumn 2009 **[see **Supporting Information—Fig. S3** ]**. The chlorophyll content decreased in all four species during the autumn but in different manners (Figs 1‒4). The chlorophyll contents of *S. aucuparia* leaflets slowly decreased during the whole measurement time, but 3‒5 days prior to abscission, the rate of degradation increased (Fig. 1A). Analysis of the earlier destructive measurements reveals similar behaviour, as the chlorophyll contents of most *S. aucuparia* leaflets decreased slowly while a few leaflets had a low chlorophyll content **[see **Supporting Information—Fig. S3** ]**.

In contrast to *S. aucuparia*, virtually no decrease in chlorophyll content occurred in any individual leaves of the other measured trees before the onset of fast chlorophyll degradation that led to the abscission of the leaf (Figs 2‒4). In *A. platanoides* (Fig. 2A) and *P. padus* (Fig. 4A), the fast degradation of chlorophyll started in a concerted manner in all studied leaves (on 13 October 2015). *Acer platanoides* leaves were detached 8‒10 days and *B. pendula* leaves 6 days after the onset of rapid chlorophyll degradation (Figs 2A and 3A). The data for *P. padus* are not conclusive but suggest similar timing as in *A. platanoides* (Fig. 4A).

In the leaves of *P. padus* and *B. pendula*, chlorophyll degradation begins in seemingly random order **[see **Supporting Information—Fig. S4** ]**. In the data measured during the autumn, chlorophyll degradation started at different times in different *B. pendula* leaves (Fig. 3A), and the leaves belonging to same branches did not behave similarly. On the contrary, in *S. aucuparia*, the timing of chlorophyll degradation differed between leaves but leaflets belonging to the same leaf had a more uniform timing (Fig. 1A). Lower leaves lost chlorophyll before the upper leaves (Fig. 1A). According to our observations, early signs of senescence in lower leaves is a feature shared by some but not all *S. aucuparia* trees **[see **Supporting Information—Fig. S4** ]**. *Acer platanoides* leaves used in the present study were from the lowest branches and in *A. platanoides* trees the upper, more exposed leaves are usually the first ones to turn red **[see **Supporting Information—Fig. S4** ]**.

### Autumnal changes in flavonol content depend on species and time

The flavonol contents of *S. aucuparia* leaflets slightly increased during the whole autumn and the increase seemed to speed up with decrease in the chlorophyll content (Fig. 1B). Increase in flavonols coincided with chlorophyll degradation also in *A. platanoides* and *B. pendula* (Figs 2B and 3B). In *A. platanoides* leaves, a slow constant increase in flavonol content continued for the whole autumn but the rate clearly increased a few days before chlorophyll degradation started (Fig. 2B). In *B. pendula* leaves, there was little or no increase in flavonols as long as the chlorophyll content of the leaf remained stable, but the flavonol content increased very rapidly when chlorophyll was degraded. This behaviour was especially clear during mid-autumn (Fig. 3B). In contrast to the other studied trees, the flavonol content of *P. padus* leaves did not change during the autumn (Fig. 4B). The same trend was observed when the rates of change in flavonol and chlorophyll contents in 2.5 days (calculated as a slope of a line) were plotted against each other **[see **Supporting Information—Fig. S5** ]**.

### Diurnal changes in chlorophyll and flavonol contents

**Figure 5.**
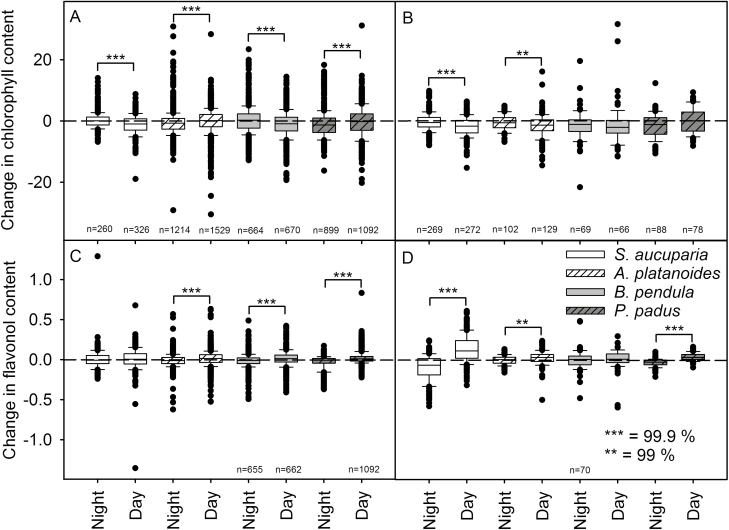
Changes in chlorophyll (A, B) or flavonol (C, D) contents during the nights (Night) or during the days (Day) in *Sorbus aucuparia* (white boxes), *Acer platanoides* (white hatched boxes), *Betula pendula* (grey boxes) and *Prunus padus* (dark grey hatched boxes) leaves. The changes are calculated for all pairs of two subsequent measurement points, separately from leaflets (*S. aucuparia*) or leaves (other species) with steady chlorophyll level (A, C) and from leaflets or leaves in which chlorophyll is being rapidly degraded (B, D). The boxes show 50 % of the data (the line indicates median), error bars show 10th and 90th percentiles, and outliers are drawn as dots. Asterisks indicate the cases where changes during the nights and changes during the days differ significantly with 99.9 % (***) or 99 % (**) probability, calculated with Mann–Whitney *U* test. Sample sizes are written below each box plot and they are similar for chlorophyll and flavonol measurements except in the case of *P. padus* in (C).

To analyse diurnal changes in chlorophyll or flavonol contents, we calculated how much the amounts of these compounds changed during the nights or the days (by subtracting the evening value from the value of the next morning and the morning value from the value measured next evening; Fig. 5). The analysis was done for all the individual leaves or leaflets of the four trees, separately for those measurements from leaves or leaflets with stable chlorophyll content and for measurements from leaves with decreasing chlorophyll content.

On the average, the chlorophyll contents of *S. aucuparia* leaflets with fairly stable chlorophyll levels did not change during the nights (Fig. 5A), whereas the change in the chlorophyll content during the days was negative, indicating that the rate of chlorophyll degradation exceeded the rate of synthesis (Fig. 5A). The difference between day and night was statistically significant, suggesting that in *S. aucuparia*, net degradation of chlorophyll occurs only during daytime. The daytime degradation led to the net loss of chlorophyll as, in contrast to the other species, the chlorophyll content of *S. aucuparia* leaves slowly decreased for the whole autumn (Fig. 1 and **Supporting Information—Fig. S3**). Also in *B. pendula* leaves with stable chlorophyll content, the average changes in chlorophyll levels during the nights and the days were significantly different, in this case suggesting chlorophyll degradation during the days (Fig. 5A). In *A. platanoides* and *P. padus* leaves with stable chlorophyll content, chlorophyll content seemed to slightly decrease during the nights (Fig. 5A).

In those *S. aucuparia* and *A. platanoides* leaves in which fast chlorophyll degradation had commenced, the loss of chlorophyll took place in the daytime (Fig. 5B). In contrast, in *P. padus* and *B. pendula* leaves with fast chlorophyll degradation, the chlorophyll content decreased similarly during the nights and the days (Fig. 5B). Loss of chlorophyll both during days and nights can also be seen in individual leaves of *B. pendula* and *P. padus* (Figs 3 and 4).

In *A. platanoides*, *B. pendula* and *P. padus* leaves with stable chlorophyll concentration, the flavonol content did not, on the average, change during the nights but increased during the days (Fig. 5C). Flavonol content increased during the days also in the leaves with decreasing chlorophyll content in all the species except that for *B. pendula* the difference was not statistically significant (Fig. 5D), suggesting that in general, flavonols are synthetized during the daytime.

## Discussion

### Chlorophyll is mainly degraded during the last week before abscission

The present data suggest that there are at least two ways to senesce: chlorophyll content of a leaf stays high for the whole autumn and starts to decrease only 5‒10 days prior to abscission (in *A. platanoides*, *B. pendula* and *P. padus*; Figs 2‒4), or chlorophyll is slowly degraded during the whole autumn (in *S. aucuparia*; Fig. 1). Even in the second case, the rate of chlorophyll degradation increases 3‒5 days before abscission of the leaflet. It has been proposed that the competence to senesce, in terms of the enzymatic machinery, might already be ready in green leaves (Andersson *et al.* 2004; Fracheboud *et al.* 2009), and that rapid senescence is advantageous for a tree (Fracheboud *et al.* 2009). Our data reveal that the loss of chlorophyll, a sign of the implementation of senescence in an individual leaf, takes only 1‒2 weeks before abscission, even though the autumn lasts 1‒3 months.

### Depending on species, chlorophyll is degraded either during the day or at all times

The circadian cycle regulates many photosynthesis-related functions of plants (e.g. Noordally *et al.* 2013), including the expression of many genes of LHC (Kloppstech 1985; Xu and Johnson 2001). The present study suggests that in *S. aucuparia* and *B. pendula* chlorophyll decreases during the day rather than the night in those leaves in which rapid chlorophyll degradation had not yet commenced (Fig. 5A). The chlorophyll content of an individual leaf may fluctuate both up and down in the autumn because the chlorophylls are synthesized and degraded in an active leaf. Chlorophyll turnover is partially associated with the repair cycle of PSII (Feierabend and Dehne 1996; Lin *et al.* 2014), in good agreement with the finding that the turnover rate increases in high light (Beisel *et al.* 2010). The half-life of chlorophyll (64.1 h in mature wheat leaves; Stobart and Hendry 1984), is short enough to allow small circadian fluctuations of the chlorophyll content.

Chlorophyll is synthesized in the daytime in angiosperms (e.g. Pan *et al.* 2015) because protochlorophyllide oxidoreductase is active only in the light (for review, see Masuda and Takamiya 2004). Diurnal fluctuations in leaf chlorophyll content can also be caused by circadian oscillations in amounts or activities of other enzymes required in chlorophyll synthesis (Papenbrock *et al.* 1999). Our data confirm that fluctuations in chlorophyll contents of tree leaves are small (see Reddy *et al.* 2000).

Diurnal variation in the rate of chlorophyll degradation during senescence has not been earlier studied. It could be argued that it is beneficial for plants to degrade chlorophyll during the night when the phototoxic intermediates of chlorophyll degradation would be less dangerous. However, in *S. aucuparia* and in *A. platanoides*, more chlorophyll was lost during the days than during the nights (Fig. 5B) and in *B. pendula* and *P. padus* there were no significant day/night differences in the rate of chlorophyll degradation (Fig. 5B). Thus, our data indicate that in spite of possible production of singlet oxygen during daytime, chlorophyll degradation is not confined to the dark period in the studied plants, but instead these species degrade chlorophyll in the light, probably relying on ROS-protection mechanisms like early light induced proteins (Andersson *et al.* 2004; Heddad *et al.* 2006).

### Chlorophyll *a/b* ratio stays stable nearly to abscission time

In our data, chlorophyll *a/b* ratio stayed constant in the senescing leaves of all four trees of different species until chlorophyll was degraded to less than 10 µg Chl cm^−2^**[see **Supporting Information—Fig. S6** ]**. This is consistent with the hypothesis that plants keep the photosynthetic machinery functional until a very late stage of senescence (Keskitalo *et al.* 2005; Moy *et al.* 2015). When the chlorophyll content had reached the limit, the ratio decreased in *A. platanoides* and *S. aucuparia*. Decrease in the chlorophyll *a/b* ratio, resulting from slower degradation of chlorophyll *b* compared to *a*, has been observed in many senescing trees (Wolf 1956; Lee *et al.* 2003; Castro and Sanchez-Azofeifa 2008). Decrease of the chlorophyll *a/b* ratio may indicate that PSI is degraded faster than PSII (Andersson *et al.* 2004; Moy *et al.* 2015) or that LHCII stays intact longer than the reaction centres (Lepeduš *et al.* 2010; Moy *et al.* 2015). In our data, chlorophyll *a/b* ratio increased in *P. padus***[see **Supporting Information—Fig. S6** ]**, which might be caused by higher stability of PSI compared to PSII, as is common in some annual species (e.g. Yamazaki *et al.* 1999). In senescing *Fagus sylvatica*, the chlorophyll *a/b* ratio first slightly increases and then decreases (Kraj 2015).

### What is the role of enhanced flavonol synthesis?

In those *B. pendula* leaves that lost their chlorophyll around 1 October, flavonols increased more than in leaves that stayed green longer (Fig. 3), and light intensities during the later days were lower (see Table 1 and **Supporting Information—Fig. S1**). Also in *S. aucuparia*, rapid flavonol accumulation seemed to occur during bright days (Table 1), suggesting that high light enhances flavonol synthesis. However, the relationship between brightness of the day and flavonol synthesis did not hold for data collected during the early autumn (Table 1). Synthesis of flavonols also takes place in the daytime (Fig. 5C and D).

**Table 1.** Night and day temperature, total irradiance (sum) and maximum daily light intensity (max), and flavonol synthesis in rowan and silver birch leaves during selected days during the autumn. Increase in flavonols during the respective days is shown with symbols, where 0 represents no change in flavonol levels and +++ represents the highest observed increase.

Species		*T* (night), °C	*T* (day), °C	Light (sum), kJ m^−2^ day^−1^	Light (max), kJ m^−2^ h^−1^	Flv
*Sorbus aucuparia*	25.8	16	27	16525	2300	+
	27.8	14	20	10641	1447	++
	23.9	12	15	5469	868	+
	25.9	11	15	5060	778	+
*Betula pendula*	31.8	9	21	17721	2146	0
	2.9	14	13	2766	270	+
	28.9	9	13	8737	1375	+++
	30.9	11	17	10071	1631	+++
	19.10	1	11	6555	1130	+
	21.10	3	10	2956	742	++

It has been previously shown in birch trees that certain flavonols increase in response to UV-B radiation (e.g. Morales *et al.* 2010). Because flavonols do not absorb UV-B as efficiently as other flavonoids (Pollastri and Tattini 2011), Majer *et al.* (2014) concluded that the differences in flavonol composition between sun and shade leaves of *Tilia platyphyllos* do not contribute to UV screening, but instead flavonols function as quenchers of singlet oxygen in strong visible light. Due to the short diffusion distance of singlet oxygen (for review, see Mattila *et al.* 2015), only flavonols present in the cytoplasm may efficiently protect the cells by reacting with this harmful species. Chlorophyll degradation may enhance flavonol synthesis because leaves that contain less chlorophyll are more efficiently illuminated throughout the leaf tissue than heavily pigmented leaves. Enhancement of photoinhibition of PSII by loss of chlorophyll (Pätsikkä *et al.* 2002) is an example of an analogous situation.

Flavonols have also been shown to increase in response to cold in dwarf birch (Stark *et al.* 2015), and flavonol content correlated with cold tolerance in hybrid *Arabidopsis thaliana* (Korn *et al.* 2008). Here, when the first *S. aucuparia* leaves fell off, the weather was warmer than later in August; however, flavonols increased at least equally rapidly in the leaves that fell off first and in those lasting longer (Fig. 1 and Table 1). Furthermore, weather was not particularly cold when flavonols strongly increased in *B. pendula* in mid-autumn (Fig. 3 and Table 1), and no additional synthesis of flavonols was observed in non-senescing leaves in October when the night temperature fell below zero. However, more data would be needed to confirm the generality of these findings.

In addition to their suggested roles in ROS-scavenging, flavonols might also function in protection against pathogens or herbivores (Martemyanov *et al.* 2015; Yang *et al.* 2016). However, birch leaves with intense yellow colour have been shown to attract more aphids than greener leaves (Sinkkonen *et al.* 2012), and larvae feeding on birch leaves with a lower chlorophyll content are not more conspicuous to birds than larvae on control leaves (Koski *et al.* 2017). Some flavonols are toxic also to plants (e.g. Kandil *et al.* 2004) and flavonols of the falling leaves might suppress the growth of competitors under the tree.

## Conclusions

It may take over 2 months for a tree to shed all its leaves during autumn senescence, but our data suggest that most of this time, individual leaves retain a relatively high chlorophyll content and the average chlorophyll content decreases only because some leaves lose chlorophyll rapidly. For that reason, a complete picture about tree senescence cannot be achieved by studying senescence as an averaged phenomenon. The rate of chlorophyll degradation, once it commences, is quite uniform in a tree. Thus, trees keep photosynthetically active leaves alongside with leaves from which nitrogen is rapidly remobilized before abscission. Constant slow decrease in chlorophyll content during the whole autumn observed in *S. aucuparia* suggests that senescence strategies vary with species. The present data also suggest that increase in flavonols is common during senescence, but their role still needs to be resolved.

## Sources of Funding

Our work was funded by Turku University Foundation (Finland; 12353 to H.M.), University of Turku Graduate School (Finland; to H.M., V.H.), Vilho, Yrjö ja Kalle Väisälän rahasto (Finland; to H.M.), Finnish Cultural Foundation (00150207 to V.H.), Academy of Finland (307335 to E.T.), Ministry of Education, Youth and Sports within the National Programme for Sustainability (Czech Republic; LO1415 to K.B.M.) and Czech Science Foundation (13-28093S to K.B.M.).

## Contributions by the Authors

H.M., D.V., V.H., S.K., O.V. and M.A. did the experiments, K.B.M. provided help and guidance with methods and E.T. supervised the project. H.M. wrote the paper with contributions from all authors.

## Conflict of Interest

None declared.

## Supporting Information

The following additional information is available in the online version of this article—

**Table S1.** The effect of the measurer (five persons) on the chlorophyll or flavonol (Flv) levels in the four species, calculated by ANOVA. A statistically significant difference is highlighted with ***.

**Figure S1.** Weather in Turku, Finland, 24 August‒21 October 2015. The grey area shows total irradiance (kJ m^−2^ day^−1^) and the black dashed line shows maximum daily light intensity (as kJ m^−2^ h^−1^). Day temperature is calculated as an average temperature at 1200‒1600 h (from 1 October onwards 1300‒1600 h), and night temperature as an average temperature at 0400‒0600 h (except before 1 August, 0200‒0400 h and after 12 October 0500‒0700 h). The data are from the Finnish Meteorological Institute.

**Figure S2.** Phenolic compounds detected with high-performance liquid chromatography (HPLC) at 355 nm from leaf extracts. (A) Chromatograms. (B‒D) Spectra of all extracted compounds of *Betula pendula* (B), *Acer platanoides* (C) and *Sorbus aucuparia* (D). Retention times (RT) and classifications of the compounds are given.

**Figure S3.** Chlorophyll content in *Sorbus aucuparia* leaves during autumn senescence. At every measurement date, 10 randomized leaflets were collected and chlorophylls were extracted in *N*,*N*-dimethylformamide (DMF) and quantified spectrophotometrically. Mean values and error bars showing SD (A); individual measurements (B).

**Figure S4.** Senescing *Sorbus aucuparia* (A, B), *Acer platanoides* (C), *Betula pendula* (D) and *Prunus padus* (E) trees.

**Figure S5.** Rates of changes in chlorophyll contents from *Sorbus aucuparia* (A), *Acer platanoides* (B), *Betula pendula* (C) and *Prunus padus* (D) leaves plotted against rates of changes in flavonol contents. Rates during 2.5 days are calculated as slopes of lines from slightly smoothed data.

**Figure S6.** Chlorophyll *a* to *b* ratio as a function of total chlorophyll concentration of the sample in the four species, measured during different phases of autumn senescence. Chlorophylls were extracted in *N*,*N*-dimethylformamide (DMF) and quantified spectrophotometrically.

**Figure S7.** Comparison of leaf chlorophyll content measured with a destructive method by extracting pigments in *N*,*N*-dimethylformamide (DMF) or with non-destructive measurements from *Sorbus aucuparia* (A), *Acer platanoides* (B), *Betula pendula* (C) and *Prunus padus* (D) leaves, collected at different dates during autumn.

**Figure S8.** The total amount (relative units) of all compounds detected with high-performance liquid chromatography (HPLC) (circles) or those compounds identified as flavonols by their spectra (squares), from extracts of *Sorbus aucuparia* leaflets (A), *Acer platanoides* leaves (B) and *Betula pendula* leaves (C), compared to the flavonol index measured from the same leaves with Dualex.

**Figure S9.** Absorbance at 355 nm, reflecting the amount of each individual compound detected with high-performance liquid chromatography (HPLC) after extraction of phenolics from leaves of *Betula pendula* (A), *Acer platanoides* (B) and *Sorbus aucuparia* (C) compared to the flavonol index measured from the same leaves with Dualex. The compounds are classified to six groups. The numbers indicate the chlorophyll content of the sample as µg Chl m^−2^.

**Figure S10.** Effect of ambient light (A, B) and handling (C) on the measuring accuracy of Dualex. (A, B) Chlorophyll and flavonol contents were measured from the same *Acer platanoides* and *Prunus padus* leaves in the presence of different amounts of incident light (photosynthetic photon flux density, PPFD, of 0‒100 µmol m^−2^ s^−1^). (C) Chlorophyll (hatched bars) and flavonol (grey bars) contents of *P. padus* leaves were measured in darkness so that the measuring heads of Dualex were either kept loosely together or pressed strongly together. The averages (*n* = 6) are significantly different with 99.9 % (***) or 95 % (*) probability, calculated with Student’s *t*-test. The error bars show SD calculated from 3‒6 technical repetitions and they are shown when larger than the symbols.

Supporting Information

## Appendix 1: Validation of the Optical Method for Chlorophyll and Flavonol Measurements from Senescing Leaves

To validate chlorophyll measurements from senescing leaves with a fluorescence and transmittance based sensor (Dualex Scientific™), we compared the chlorophyll content measured with the non-destructive method with extraction of chlorophylls in *N*,*N*-dimethylformamide (DMF). The results obtained with the two methods showed linear correlation (*R*^2^ = 0.8‒0.96) in a wide range of chlorophyll concentrations **[see **Supporting Information—Fig. S7** ]**. In agreement with earlier literature (Cerovic *et al.* 2012; Casa *et al.* 2015; Agati *et al.* 2016), species-specific calibration was found to be necessary for quantitative measurements **[see **Supporting Information—Fig. S7** ]**.

To validate the non-destructive flavonol measurements from senescing leaves, flavonols were also measured with high-performance liquid chromatography (HPLC) **[see **Supporting Information—Fig. S8** ]**. In the case of senescing leaves, a good correlation between the optical method and HPLC was obtained in *Betula pendula* leaves, although the relationship is somewhat curvilinear **[see **Supporting Information—Fig. S8C** ]**. The reason for the good correlation in *B. pendula* might be that this species does not synthetize anthocyanins in contrast to *Sorbus aucuparia* and *Acer platanoides*. Accordingly, large amounts of phenolic acids and non-flavonol flavonoids were observed in *S. aucuparia* and *A. platanoides*, and many of these compounds also increased during senescence **[see **Supporting Information—Fig. S9** ]**, but the optical method correlated with both the amount of flavonols and with the total amount of phenolic compounds **[see **Supporting Information—Fig. S8** ]**. A good correlation with the amounts of flavonols in the leaf measured with the optical method and with HPLC is in accordance with previous research (Barthod *et al.* 2007; Pfündel *et al.* 2007; Agati *et al.* 2016), although the optical method is expected to measure epidermal compounds, whereas extraction methods measure an average of the whole leaf. However, flavonols cannot be distinguished from other phenolic compounds with the optical method. In our case, this difficulty has only minor importance because relative differences in the amounts of flavonols in leaves at different stages of senescence were highly correlated with relative differences in the amounts of all phenolic compounds **[see **Supporting Information—Fig. S8** ]**.

We conducted the measurements in field conditions, either at sunrise or at sunset, both defined by the position of half of the Sun above the horizon. Ambient light was found not to affect chlorophyll measurements **[see **Supporting Information—Fig. S10A** ]**. Flavonol values showed some variability with ambient light **[see **Supporting Information—Fig. S10B** ]**, and high incident light (photosynthetic photon flux density ~100 µmol m^−2^ s^−1^) occasionally caused an inability to record any value. However, no consistent dependence of the flavonol signal on ambient light was observed.

We found that pressing the measuring heads of the Dualex device more firmly together slightly lowered the observed chlorophyll value and increased the flavonol value **[see **Supporting Information—Fig. S10C** ]**, suggesting that the results might depend on the measurer. However, according to ANOVA analysis, the measurer (five persons here) did not have a significant effect on the chlorophyll or flavonol levels, except for the case of the chlorophyll content of *A. platanoides***[see **Supporting Information—Table S1** ]**. Even though the assumptions of ANOVA are not met in the present data, the result suggests that the effect of the measurer is small.

## References

[CIT0001] Agati G, Tuccio L, Kusznierewicz B, Chmiel T, Bartoszek A, Kowalski A, Grzegorzewska M, Kosson R, Kaniszewski S 2016 Nondestructive optical sensing of flavonols and chlorophyll in white head cabbage (*Brassica oleracea* L. var. capitata subvar. alba) grown under different nitrogen regimens. Journal of Agricultural and Food Chemistry 64:85–94. 26679081 10.1021/acs.jafc.5b04962

[CIT0002] Andersson A, Keskitalo J, Sjödin A, Bhalerao R, Sterky F, Wissel K, Tandre K, Aspeborg H, Moyle R, Ohmiya Y, Bhalerao R, Brunner A, Gustafsson P, Karlsson J, Lundeberg J, Nilsson O, Sandberg G, Strauss S, Sundberg B, Uhlen M, Jansson S, Nilsson P 2004 A transcriptional timetable of autumn senescence. Genome Biology 5:R24. 15059257 10.1186/gb-2004-5-4-r24 PMC395783

[CIT0003] Archetti M 2009a Classification of hypotheses on the evolution of autumn colours. Oikos 118:328–333.

[CIT0004] Archetti M 2009b Phylogenetic analysis reveals a scattered distribution of autumn colours. Annals of Botany 103:703–713. 19126636 10.1093/aob/mcn259 PMC2707868

[CIT0005] Barthod S, Cerovic Z, Epron D 2007 Can dual chlorophyll fluorescence excitation be used to assess the variation in the content of UV-absorbing phenolic compounds in leaves of temperate tree species along a light gradient?Journal of Experimental Botany 58:1753–1760. 17404380 10.1093/jxb/erm030

[CIT0006] Beisel KG, Jahnke S, Hofmann D, Köppchen S, Schurr U, Matsubara S 2010 Continuous turnover of carotenes and chlorophyll a in mature leaves of *Arabidopsis* revealed by ^14^CO_2_ pulse-chase labeling. Plant Physiology 152:2188–2199. 20118270 10.1104/pp.109.151647 PMC2850008

[CIT0007] Bhalerao R, Keskitalo J, Sterky F, Erlandsson R, Björkbacka H, Birve SJ, Karlsson J, Gardeström P, Gustafsson P, Lundeberg J, Jansson S 2003 Gene expression in autumn leaves. Plant Physiology 131:430–442. 12586868 10.1104/pp.012732 PMC166820

[CIT0008] Brant AN, Chen HYH 2015 Patterns and mechanisms of nutrient resorption in plants. Critical Reviews in Plant Sciences 34:471–486.

[CIT0009] Christ B, Hörtensteiner S 2014 Mechanism and significance of chlorophyll breakdown. Journal of Plant Growth Regulation 33:4–20.

[CIT0010] Casa R, Castaldi F, Pascucci S, Pignatti S 2015 Chlorophyll estimation in field crops: an assessment of handheld leaf meters and spectral reflectance measurements. Journal of Agricultural Science 153:876–890.

[CIT0011] Castro KL, Sanchez-Azofeifa GA 2008 Changes in spectral properties, chlorophyll content and internal mesophyll structure of senescing *Populus balsamifera* and *Populus tremuloides* leaves. Sensors 8:51–69. 27879696 10.3390/s8010051 PMC3681139

[CIT0012] Cerovic ZG, Masdoumier G, Ghozlen NB, Latouche G 2012 A new optical leaf-clip meter for simultaneous non-destructive assessment of leaf chlorophyll and epidermal flavonoids. Physiologia Plantarum 146:251–260. 22568678 10.1111/j.1399-3054.2012.01639.x PMC3666089

[CIT0013] Collier DE, Thibodeau BA 1995 Changes in respiration and chemical content during autumnal senescence of *Populus tremuloides* and *Quercus rubra* leaves. Tree Physiology 15:759–764. 14965995 10.1093/treephys/15.11.759

[CIT0014] Falcone Ferreyra ML, Rius SP, Casati P 2012 Flavonoids: biosynthesis, biological functions, and biotechnological applications. Frontiers in Plant Science 3:222. 23060891 10.3389/fpls.2012.00222 PMC3460232

[CIT0015] Feierabend J, Dehne S 1996 Fate of the porphyrin cofactors during the light-dependent turnover of catalase and of the photosystem II reaction-center protein D1 in mature rye leaves. Planta 198:413–422.

[CIT0015a] Fischer AM 2012 The complex regulation of senescence. Critical Reviews in Plant Sciences 31:124–147.

[CIT0016] Fu YSH, Campioli M, Vitasse Y, De Boeck HJ, Van den Berge J, AbdElgawad H, Asard H, Piao S, Deckmyn G, Janssens IA 2014 Variation in leaf flushing date influences autumnal senescence and next year’s flushing date in two temperate tree species. Proceedings of the National Academy of Sciences of the Unites States of America 111:7355–7360. 10.1073/pnas.1321727111 PMC4034254 24799708

[CIT0017] Fracheboud Y, Luquez V, Björkén L, Sjödin A, Tuominen H, Jansson S 2009 The control of autumn senescence in European aspen. Plant Physiology 149:1982–1991. 19201914 10.1104/pp.108.133249 PMC2663763

[CIT0018] Gill AL, Gallinat AS, Sanders-DeMott R, Rigden AJ, Short Gianotti DJ, Mantooth JA, Templer PH 2015 Changes in autumn senescence in northern hemisphere deciduous trees: a meta-analysis of autumn phenology studies. Annals of Botany 116: 875–888. 25968905 10.1093/aob/mcv055 PMC4640124

[CIT0019] Guo Y, Gan SS 2012 Convergence and divergence in gene expression profiles induced by leaf senescence and 27 senescence-promoting hormonal, pathological and environmental stress treatments. Plant, Cell & Environment 35:644–655. 10.1111/j.1365-3040.2011.02442.x 21988545

[CIT0020] Heddad M, Norén H, Reiser V, Dunaeva M, Andersson B, Adamska I 2006 Differential expression and localization of early light-induced proteins in *Arabidopsis*. Plant Physiology 142:75–87. 16829586 10.1104/pp.106.081489 PMC1557597

[CIT0021] Hopkins M, Taylor C, Liu Z, Ma F, McNamara L, Wang TW, Thompson JE 2007 Regulation and execution of molecular disassembly and catabolism during senescence. The New Phytologist 175:201–214. 17587370 10.1111/j.1469-8137.2007.02118.x

[CIT0022] Hörtensteiner S, Kräutler B 2011 Chlorophyll breakdown in higher plants. Biochimica Et Biophysica Acta 1807:977–988. 21167811 10.1016/j.bbabio.2010.12.007

[CIT0023] Kandil FE, Grace MH, Seigler DS, Cheeseman JM 2004 Polyphenolics in *Rhizophora mangle* L. leaves and their changes during leaf development and senescence. Trees 18: 518–528.

[CIT0024] Kasuga J, Hashidoko Y, Nishioka A, Yoshiba M, Arakawa K, Fujikawa S 2008 Deep supercooling xylem parenchyma cells of katsura tree (*Cercidiphyllum japonicum*) contain flavonol glycosides exhibiting high anti-ice nucleation activity. Plant, Cell & Environment 31:1335–1348. 10.1111/j.1365-3040.2008.01835.x 18518920

[CIT0025] Keskitalo J, Bergquist G, Gardeström P, Jansson S 2005 A cellular timetable of autumn senescence. Plant Physiology 139:1635–1648. 16299183 10.1104/pp.105.066845 PMC1310548

[CIT0026] Kloppstech K 1985 Diurnal and circadian rhythmicity in the expression of light-induced plant nuclear messenger RNAs. Planta 165:502–506. 24241223 10.1007/BF00398095

[CIT0027] Korn M, Peterek S, Mock HP, Heyer AG, Hincha DK 2008 Heterosis in the freezing tolerance, and sugar and flavonoid contents of crosses between *Arabidopsis thaliana* accessions of widely varying freezing tolerance. Plant, Cell & Environment 31:813–827. 10.1111/j.1365-3040.2008.01800.x PMC2440548 18284584

[CIT0028] Koski T-M, Lindstedt C, Klemola T, Troscianko J, Mäntylä E, Tyystjärvi E, Stevens M, Helander M, Laaksonen T 2017 Insect herbivory may cause changes in the visual properties of leaves and affect the camouflage of herbivores to avian predators. Behavioral Ecology and Sociobiology 71:97.

[CIT0029] Kraj W 2015 Chlorophyll degradation and the activity of chlorophyllase and Mg-dechelatase during leaf senescence in *Fagus sylvatica*. Dendrobiology 74:43–57.

[CIT0030] Landi M, Tattini M, Gould KS 2015 Multiple functional roles of anthocyanins in plant-environment interactions. Environmental and Experimental Botany 119:4–17.

[CIT0031] Lee DW, O’Keefe J, Holbrook NM, Feild TS 2003 Pigment dynamics and autumn leaf senescence in a New England deciduous forest, eastern USA. Ecological Research 18:677–694.

[CIT0032] Lepeduš H, Jurković V, Štolfa I, Ćurković-Perica M, Fulgosi H, Cesar V 2010 Changes in photosystem II photochemistry in senescing maple leaves. Croatica Chemica Acta 83:379–386.

[CIT0033] Lin YP, Lee TY, Tanaka A, Charng YY 2014 Analysis of an *Arabidopsis* heat-sensitive mutant reveals that chlorophyll synthase is involved in reutilization of chlorophyllide during chlorophyll turnover. The Plant Journal 80:14–26. 25041167 10.1111/tpj.12611

[CIT0034] Majer P, Neugart S, Krumbein A, Schreiner M, Hideg É 2014 Singlet oxygen scavenging by leaf flavonoids contributes to sunlight acclimation in *Tilia platyphyllos*. Environmental and Experimental Botany 100:1–9.

[CIT0035] Martemyanov VV, Pavlushin SV, Dubovskiy IM, Belousova IA, Yushkova YV, Morosov SV, Chernyak EI, Glupov VV 2015 Leaf surface lipophilic compounds as one of the factors of silver birch chemical defense against larvae of gypsy moth. PLoS One 10:e0121917. 25816371 10.1371/journal.pone.0121917 PMC4376524

[CIT0036] Masuda T, Takamiya K 2004 Novel Insights into the enzymology, regulation and physiological functions of light-dependent protochlorophyllide oxidoreductase in angiosperms. Photosynthesis Research 81:1–29. 16328844 10.1023/B:PRES.0000028392.80354.7c

[CIT0037] Mattila H, Khorobrykh S, Havurinne V, Tyystjärvi E 2015 Reactive oxygen species: reactions and detection from photosynthetic tissues. Journal of Photochemistry and Photobiology B: Biology 152:176–214. 10.1016/j.jphotobiol.2015.10.001 26498710

[CIT0038] Morales LO, Tegelberg R, Brosché M, Keinänen M, Lindfors A, Aphalo PJ 2010 Effects of solar UV-A and UV-B radiation on gene expression and phenolic accumulation in *Betula pendula* leaves. Tree Physiology 30:923–934. 20519675 10.1093/treephys/tpq051

[CIT0039] Moy A, Le S, Verhoeven A 2015 Different strategies for photoprotection during autumn senescence in maple and oak. Physiologia Plantarum 155:205–216. 25656106 10.1111/ppl.12331

[CIT0040] Noordally ZB, Ishii K, Atkins KA, Wetherill SJ, Kusakina J, Walton EJ, Kato M, Azuma M, Tanaka K, Hanaoka M, Dodd AN 2013 Circadian control of chloroplast transcription by a nuclear-encoded timing signal. Science 339:1316–1319. 23493713 10.1126/science.1230397

[CIT0041] Pan W-J, Wang X, Deng Y-R, Li J-H, Chen W, Chiang JY, Yang J-B, Zheng L 2015 Nondestructive and intuitive determination of circadian chlorophyll rhythms in soybean leaves using multispectral imaging. Scientific Reports 5:11108. 26059057 10.1038/srep11108 PMC4461922

[CIT0042] Papenbrock J, Mock HP, Kruse E, Grimm B 1999 Expression studies in tetrapyrrole biosynthesis: inverse maxima of magnesium chelatase and ferrochelatase activity during cyclic photoperiods. Planta 208:264–273.

[CIT0043] Pätsikkä E, Kairavuo M, Sersen F, Aro EM, Tyystjärvi E 2002 Excess copper predisposes photosystem II to photoinhibition in vivo by outcompeting iron and causing decrease in leaf chlorophyll. Plant Physiology 129:1359–1367. 12114589 10.1104/pp.004788 PMC166529

[CIT0044] Peoples MB, Dalling MJ 1988 The interplay between proteolysis and amino acid metabolism during senescence and nitrogen reallocation. In: Noodén LD, Leopold AC, eds. Senescence and aging in plants. San Diego, CA: Academic Press, 181–217.

[CIT0045] Pfündel EE, Ghozlen NB, Meyer S, Cerovic ZG 2007 Investigating UV screening in leaves by two different types of portable UV fluorimeters reveals in vivo screening by anthocyanins and carotenoids. Photosynthesis Research 93:205–221. 17286190 10.1007/s11120-007-9135-7

[CIT0046] Pollastri S, Tattini M 2011 Flavonols: old compounds for old roles. Annals of Botany 108:1225–1233. 21880658 10.1093/aob/mcr234 PMC3197460

[CIT0047] Porra RJ, Thompson WA, Kriedemann PE 1989 Determination of accurate extinction coefficients and simultaneous equations for assaying chlorophylls *a* and *b* extracted with four different solvents: verification of the concentration of chlorophyll standards by atomic absorption spectroscopy. Biochimica et Biophysica Acta 975:384–394.

[CIT0048] Pružinská A, Anders I, Aubry S, Schenk N, Tapernoux-Lüthi E, Müller T, Kräutler B, Hörtensteiner S 2007 In vivo participation of red chlorophyll catabolite reductase in chlorophyll breakdown. The Plant Cell 19:369–387. 17237353 10.1105/tpc.106.044404 PMC1820978

[CIT0049] R Core Team 2014 R: a language and environment for statistical computing. Vienna, Austria: R Foundation for Statistical Computing.

[CIT0050] Reddy MS, Naithani S, Tuli R, Sane PV 2000 Diurnal regulation of plastid genes in *Populus deltoides*. Indian Journal of Biochemistry & Biophysics 37:453–458. 11355633

[CIT0051] Seal T 2016 Quantitative HPLC analysis of phenolic acids, flavonoids and ascorbic acid in four different solvent extracts of two wild edible leaves, *Sonchus arvensis* and *Oenanthe linearis* of North-Eastern region in India. Journal of Applied Pharmaceutical Science 6:157–166.

[CIT0052] Sinkkonen A, Somerkoski E, Paaso U, Holopainen JK, Rousi M, Mikola J 2012 Genotypic variation in yellow autumn leaf colours explains aphid load in silver birch. The New Phytologist 195:461–469. 22548444 10.1111/j.1469-8137.2012.04156.x

[CIT0053] Solovchenko AE, Merzlyak MN 2008 Screening of visible and uv radiation as a photoprotective mechanism in plants. Russian Journal of Plant Physiology 55:719–737.

[CIT0054] Springer A, Acker G, Bartsch S, Bauerschmitt H, Reinbothe S, Reinbothe C 2015 Differences in gene expression between natural and artificially induced leaf senescence in barley. Journal of Plant Physiology 176:180–191. 25637827 10.1016/j.jplph.2015.01.004

[CIT0055] Stark S, Väisänen M, Ylänne H, Julkunen-Tiitto R, Martz F 2015 Decreased phenolic defence in dwarf birch (*Betula nana*) after warming in subarctic tundra. Polar Biology 38:1993–2005.

[CIT0056] Stobart AK, Hendry GAF 1984 The turnover of chlorophyll in greening wheat leaves. Phytochemistry 23:27–30.

[CIT0057] Torras-Claveria L, Jáuregui O, Codina C, Tiburcio AF, Bastida J, Viladomat F 2012 Analysis of phenolic compounds by high-performance liquid chromatography coupled to electrospray ionization tandem mass spectrometry in senescent and water-stressed tobacco. Plant Science 182:71–78. 22118617 10.1016/j.plantsci.2011.02.009

[CIT0058] Wen CH, Lin SS, Chu FH 2015 Transcriptome analysis of a subtropical deciduous tree: autumn leaf senescence gene expression profile of formosan gum. Plant & Cell Physiology 56:163–174. 25392065 10.1093/pcp/pcu160

[CIT0059] Wolf FT 1956 Changes in chlorophyll-a and chlorophyll-b in autumn leaves. American Journal of Botany 43:714–718.

[CIT0060] Xu Y, Johnson CH 2001 A clock- and light-regulated gene that links the circadian oscillator to LHCB gene expression. The Plant Cell 13:1411–1425. 11402169 10.1105/tpc.13.6.1411 PMC135572

[CIT0061] Yamazaki J, Kamimura Y, Okada M, Sugimura Y 1999 Changes in photosynthetic characteristics and photosystem stoichiometries in the lower leaves in rice seedlings. Plant Science 148:155–163.

[CIT0062] Yang W, Xu X, Li Y, Wang Y, Li M, Wang Y, Ding X, Chu Z 2016 Rutin-mediated priming of plant resistance to three bacterial pathogens initiating the early SA signal pathway. PLoS One 11:e0146910 26751786 10.1371/journal.pone.0146910 PMC4713477

