# Supplementary material for: Degradation of chlorophyll and synthesis of flavonols during autumn senescence—the story told by individual leaves
Source: AoB Plants. 2018 May 4;10(3):ply028. doi: 10.1093/aobpla/ply028 (PMC6007487; doi:10.1093/aobpla/ply028)
Supplement: Supporting Information [file ply028_suppl_supporting_information.doc]

RESEARCH ARTICLE

**Degradation of chlorophyll and synthesis of flavonols during autumn senescence –The story told by individual leaves**

**SUPPORTING INFORMATION**

**Table S1.** The effect of the measurer (five persons) on the chlorophyll or flavonol (Flv) levels in the four species, calculated by ANOVA. A statistically significant difference is highlighted with ***.

| **Species** |  | **F value** | **Significance probability** |
| --- | --- | --- | --- |
| ***S. aucuparia*** | **Chl** | 2.66 | 0.103 |
|  | **Flv** | 0.514 | 0.473 |
| ***A. platanoides*** | **Chl** | 14.6 | 0.000143 *** |
|  | **Flv** | 0.039 | 0.844 |
| ***B. pendula*** | **Chl** | 1.26 | 0.261 |
|  | **Flv** | 0 | 0.982 |
| ***P. padus*** | **Chl** | 0.303 | 0.582 |
|  | **Flv** | 0 | 0.988 |


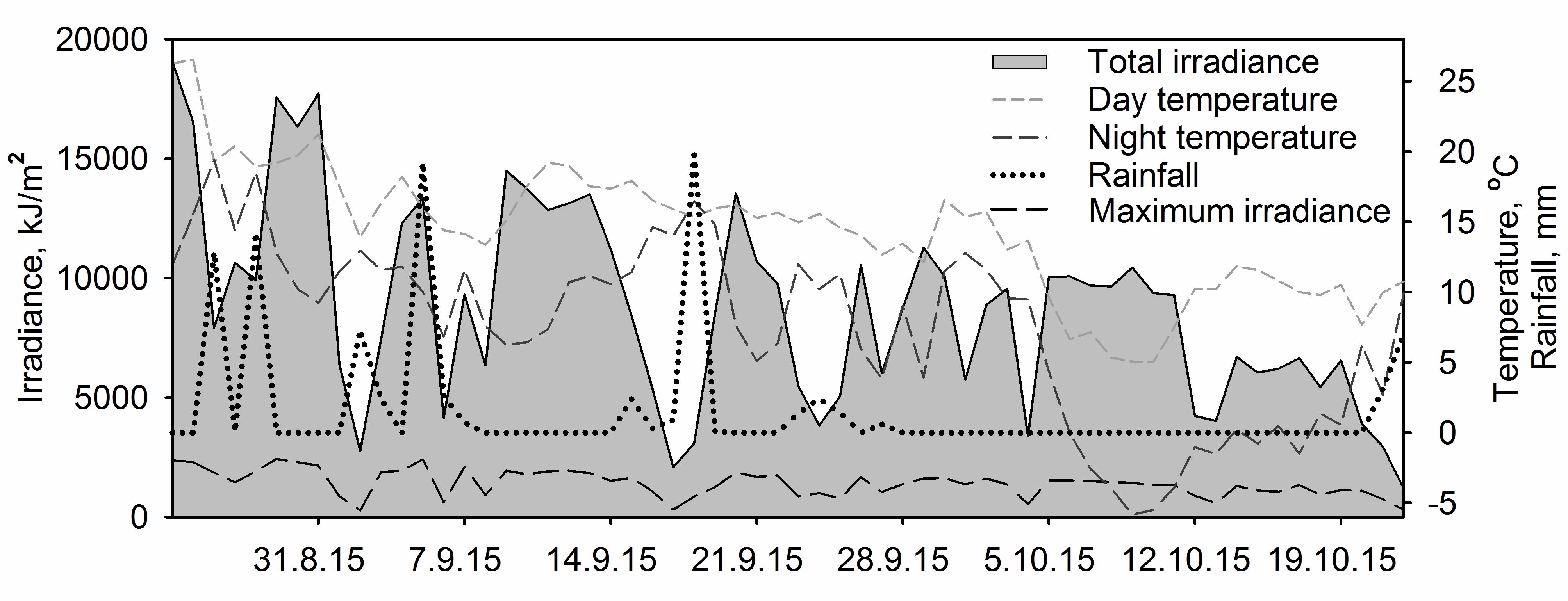


**Fig. S1.** Weather in Turku, Finland 24 August‒21 October 2015. The grey area shows total irradiance (kJ m^-2^ day^-1^) and the black dashed line shows maximum daily light intensity (as kJ m^-2^ h^-1^). Day temperature is calculated as an average temperature at 1200‒1600 h (from 1 October onwards 1300‒1600 h), and night temperature as an average temperature at 0400‒0600 h (except before 1 August, 0200‒0400 h and after 12 October 0500‒0700 h). The data are from the Finnish Meteorological Institute.


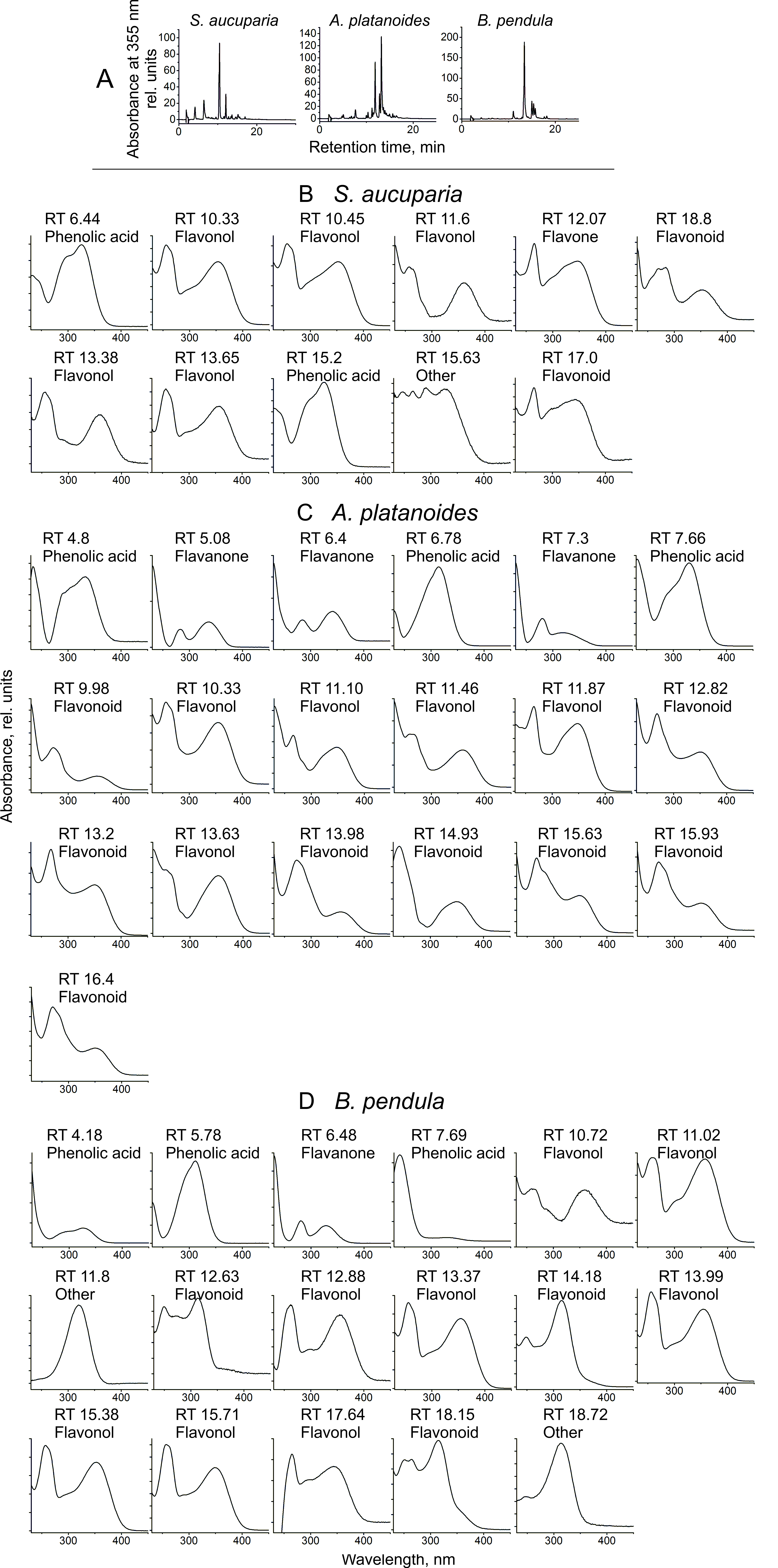


**Fig. S2.** Phenolic compounds detected with HPLC at 355 nm from leaf extracts. (A) Chromatograms. (B‒D) Spectra of all extracted compounds of *B. pendula* (B), *A. platanoides* (C) and *S. aucuparia* (D). Retention times (RT) and classifications of the compounds are given.


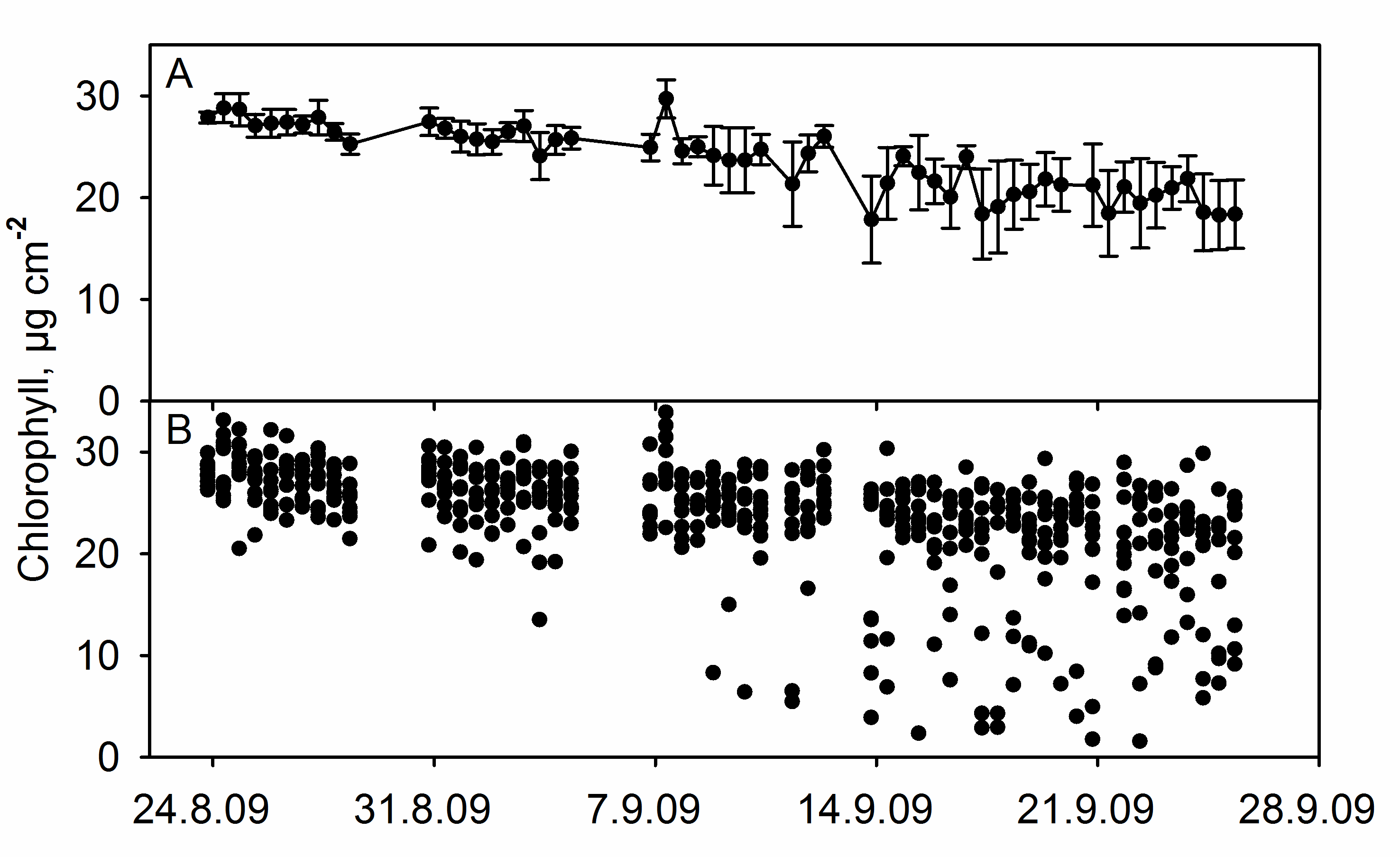


**Fig. S3.** Chlorophyll content in *S. aucuparia* leaves during autumn senescence. At every measurement date, 10 randomized leaflets were collected and chlorophylls were extracted in DMF and quantified spectrophotometrically. Mean values and error bars showing SD (A); individual measurements (B).


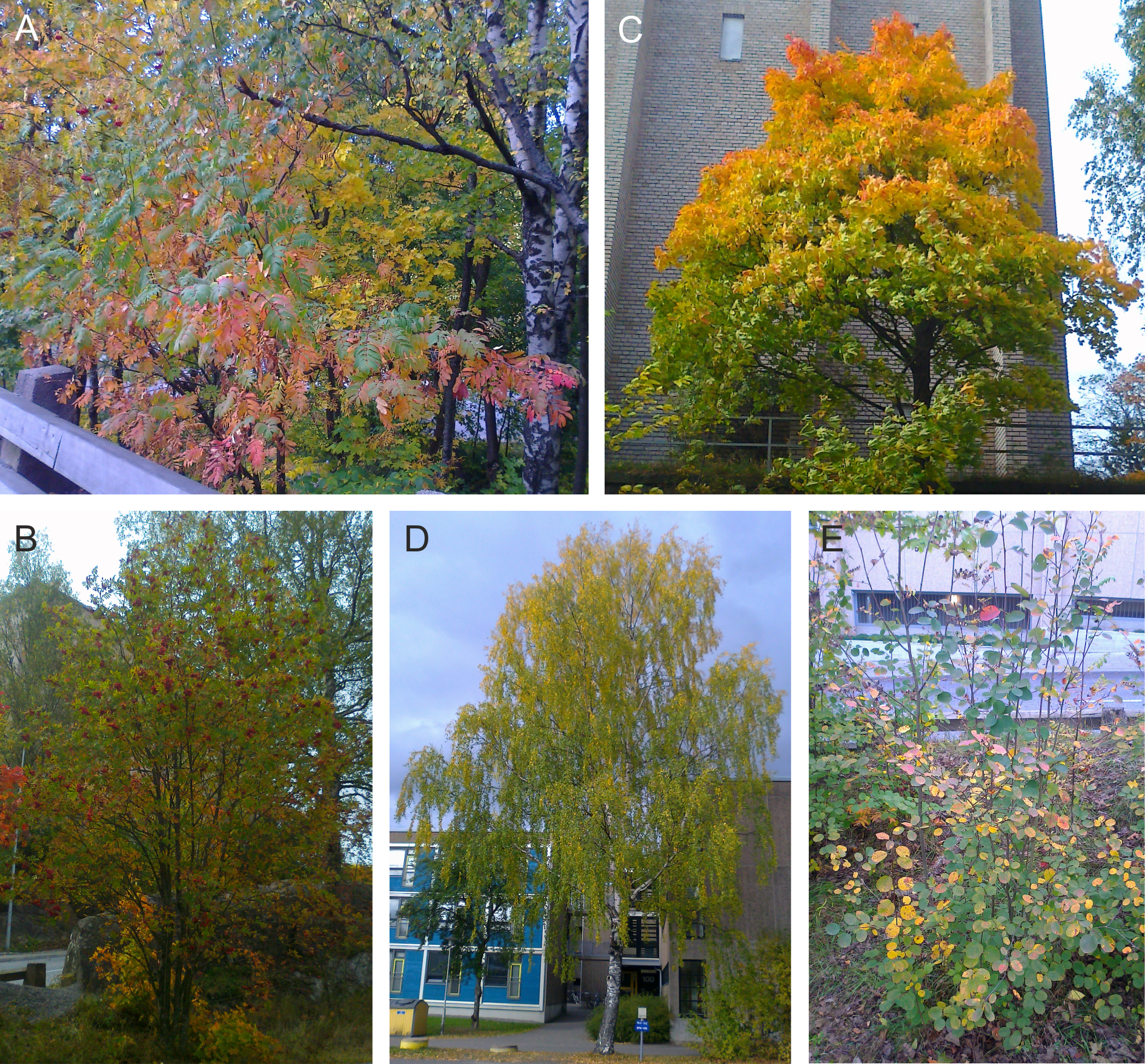


**Fig. S4.** Senescing *S. aucuparia* (A, B), *A. platanoides* (C), *B. pendula* (D) and *P. padus* (E) trees.


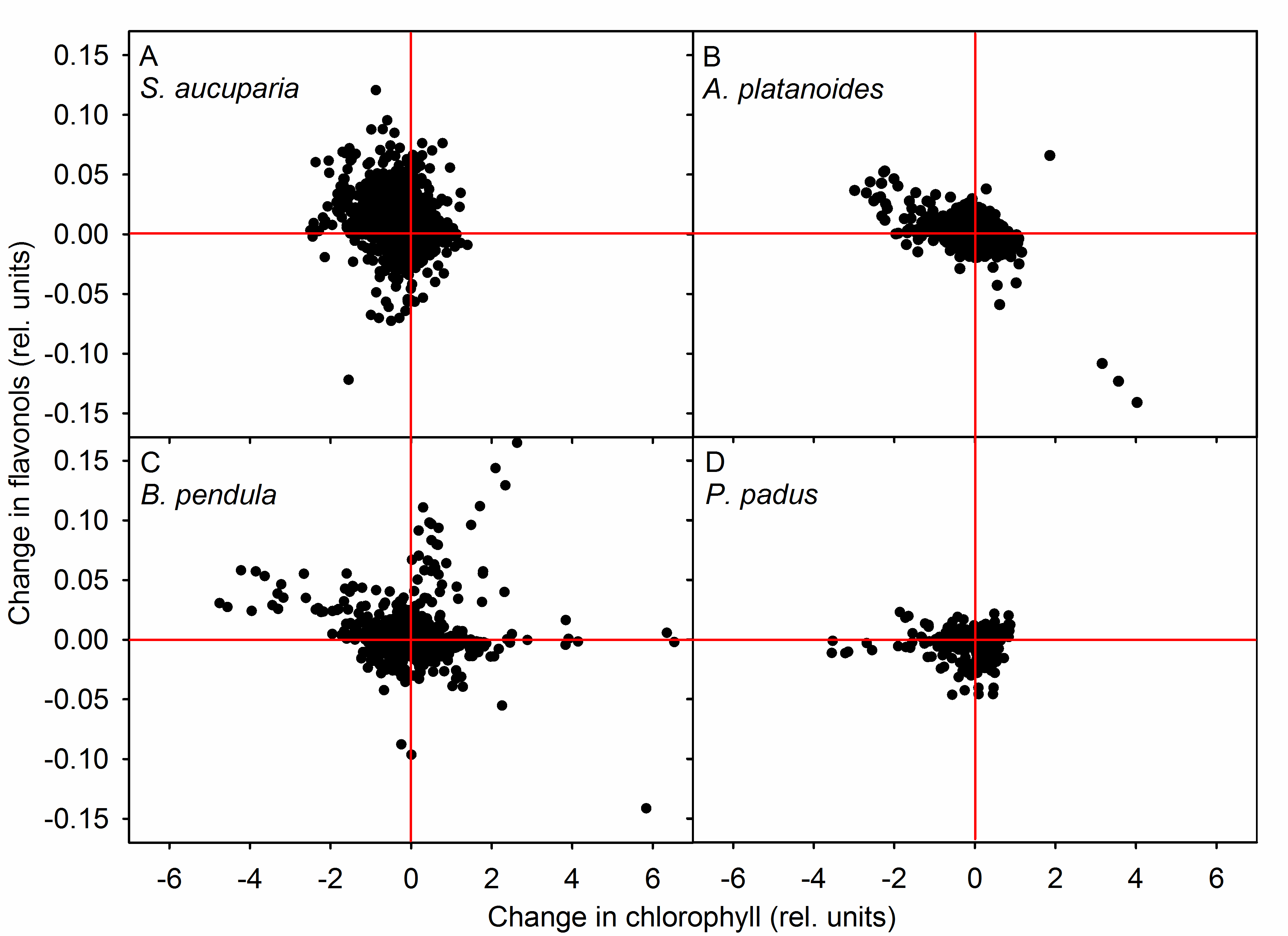


**Fig. S5.** Rates of changes in chlorophyll contents from *S. aucuparia* (A), *A. platanoides* (B), *B. pendula* (C) and *P. padus* (D) leaves plotted against rates of changes in flavonol contents. Rates during 2.5 days are calculated as slopes of lines from slightly smoothed data.


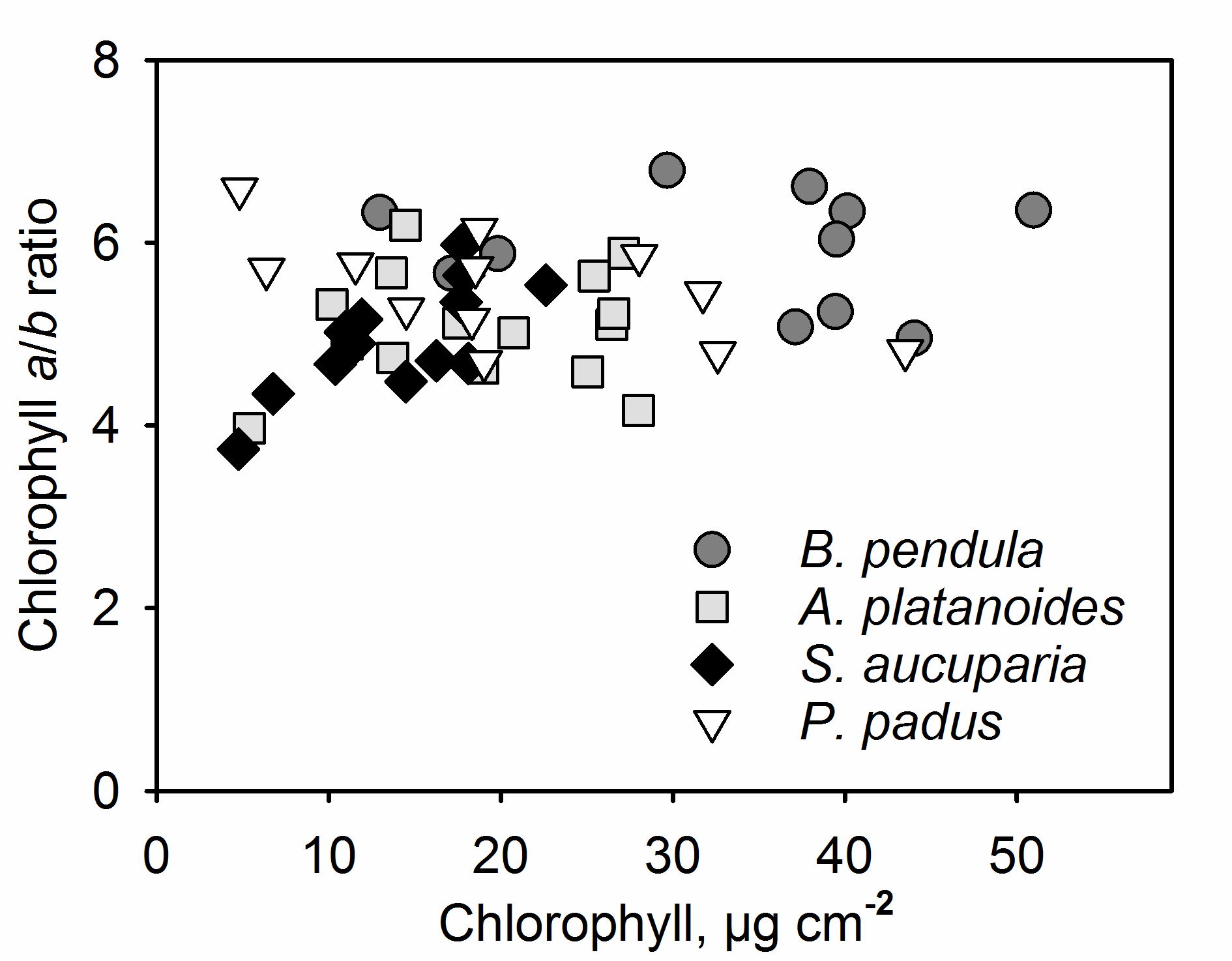


**Fig. S6.** Chlorophyll *a* to *b* ratio as a function of total chlorophyll concentration of the sample in the four species, measured during different phases of autumn senescence. Chlorophylls were extracted in DMF and quantified spectrophotometrically.


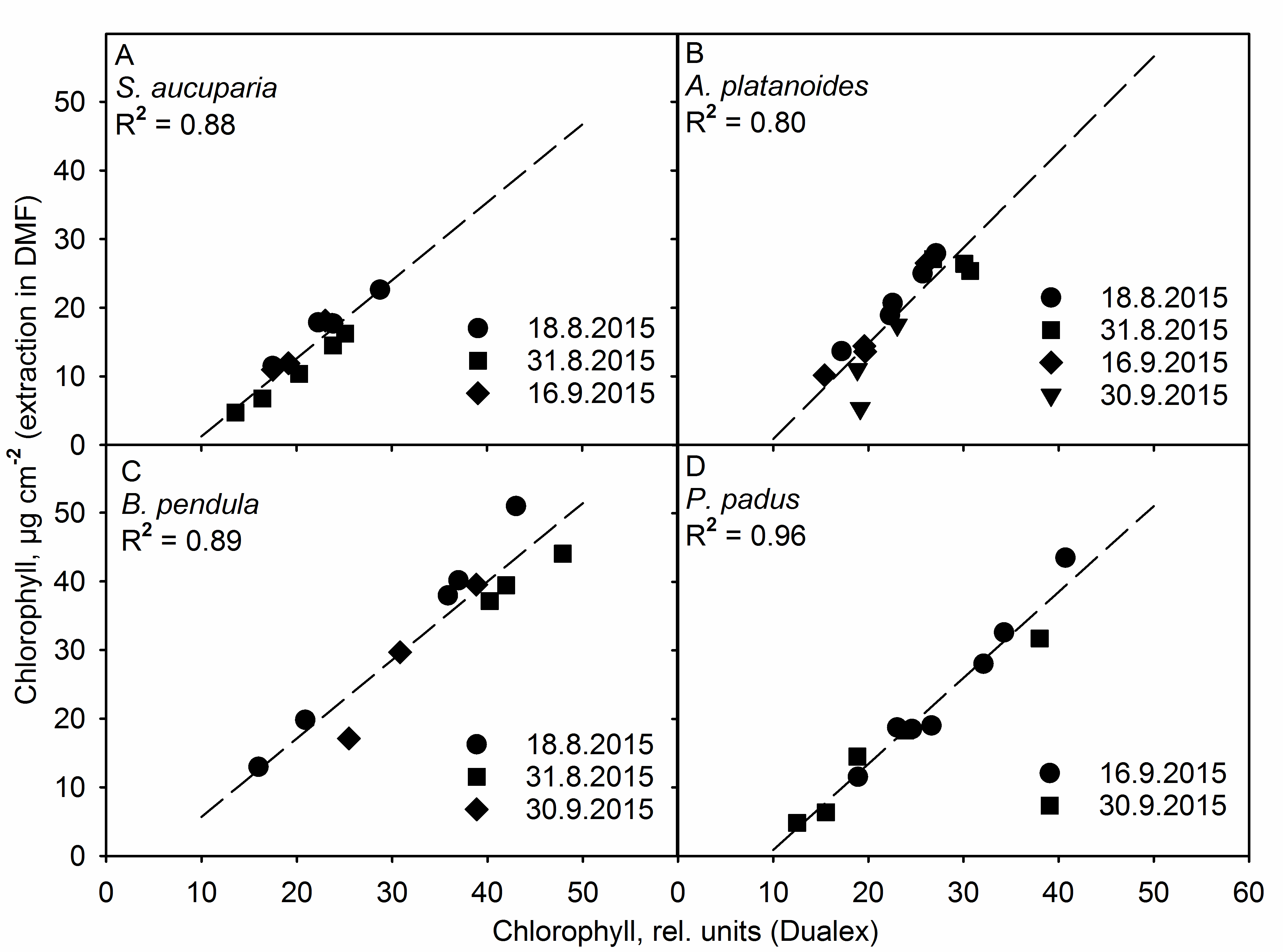


**Fig. S7.** Comparison of leaf chlorophyll content measured with a destructive method by extracting pigments in DMF or with non-destructive measurements from *S. aucuparia* (A), *A. platanoides* (B), *B. pendula* (C) and *P. padus* (D) leaves, collected at different dates during autumn.


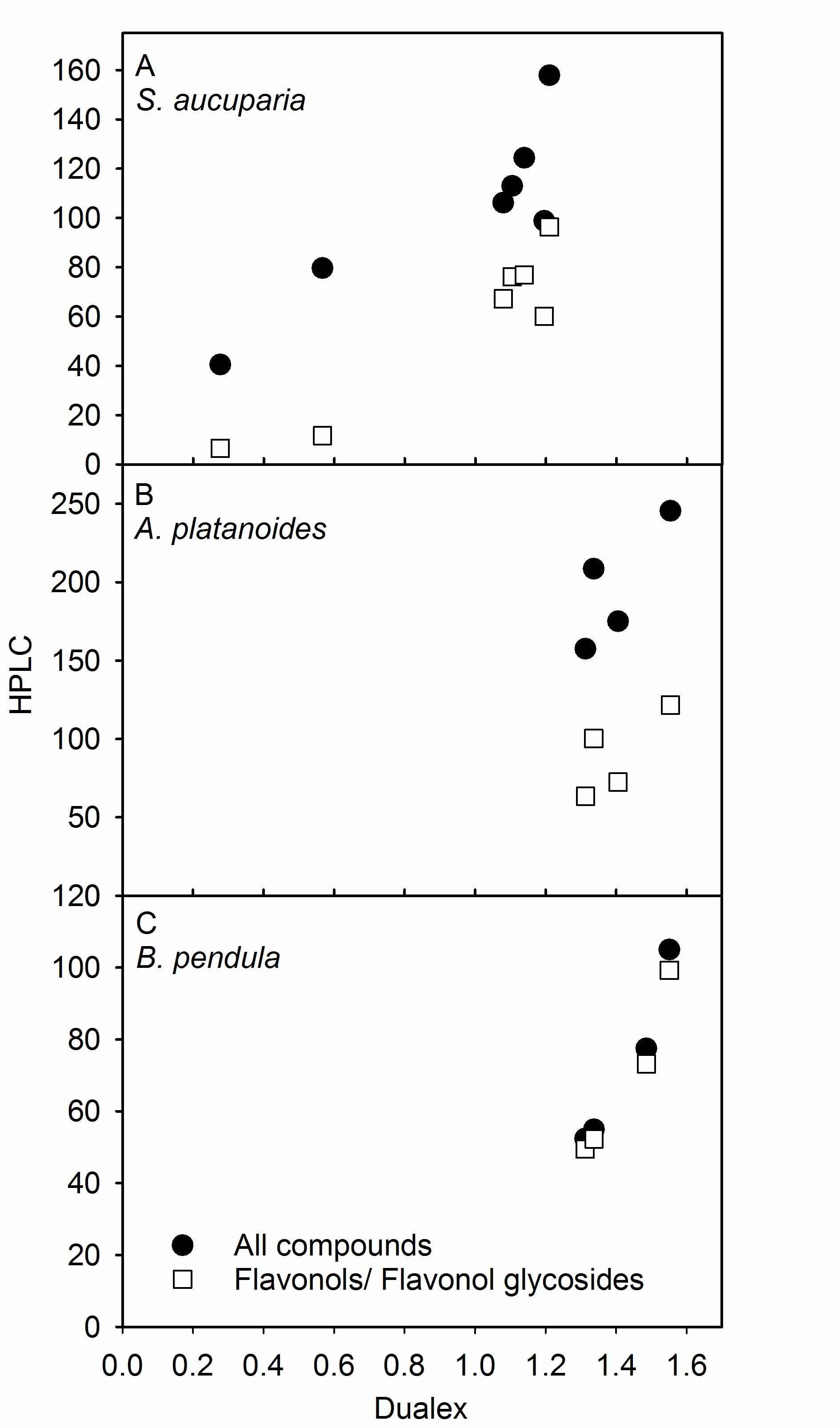


**Fig. S8.** The total amount (relative units) of all compounds detected with HPLC (circles) or those compounds identified as flavonols by their spectra (squares), from extracts of *S. aucuparia* leaflets (A), *A. platanoides* leaves (B) and *B. pendula* leaves (C), compared to the flavonol index measured from the same leaves with Dualex.


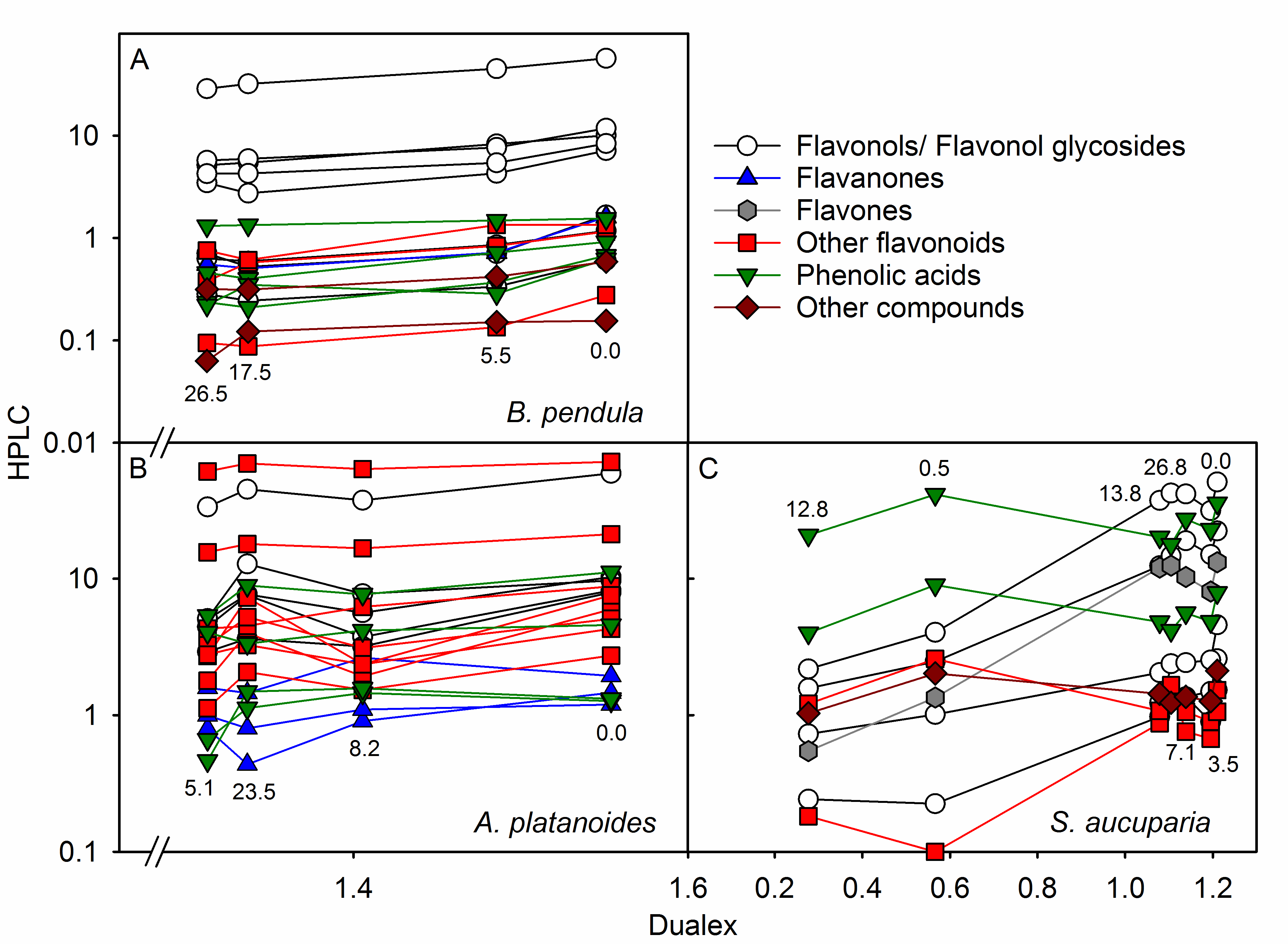


**Fig. S9.** Absorbance at 355 nm, reflecting the amount of each individual compound detected with HPLC after extraction of phenolics from leaves of *B. pendula* (A), *A. platanoides* (B) and *S. aucuparia* (C) compared to the flavonol index measured from the same leaves with Dualex. The compounds are classified to six groups. The numbers indicate the chlorophyll content of the sample as µg Chl m^-2^.


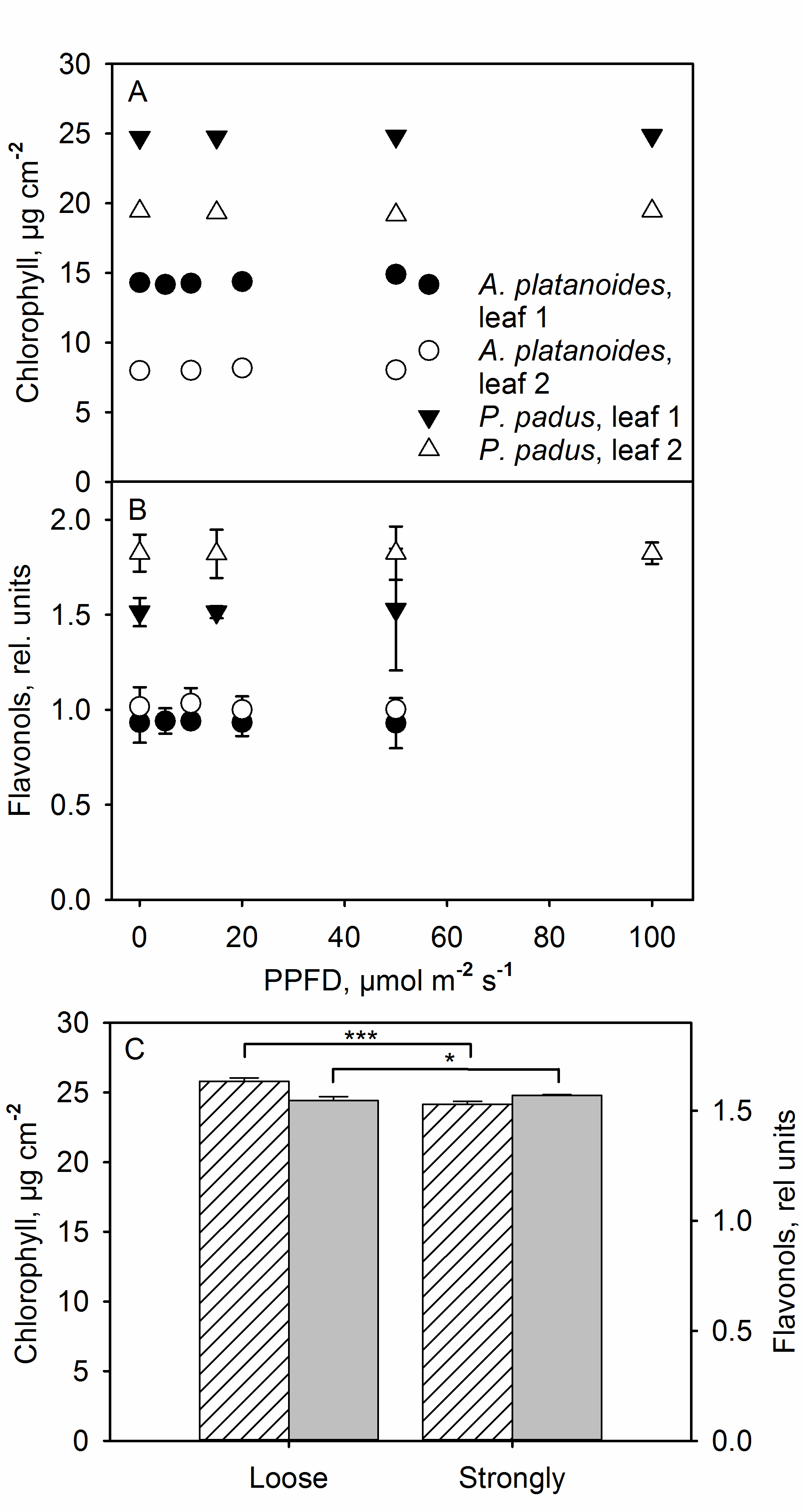


**Fig. S10.** Effect of ambient light (A, B) and handling (C) on the measuring accuracy of Dualex. (A, B) Chlorophyll and flavonol contents were measured from the same *A. platanoides* and *P. padus* leaves in the presence of different amounts of incident light (photosynthetic photon flux density, PPFD, of 0‒100 µmol m^-2^ s^-1^). (C) Chlorophyll (hatched bars) and flavonol (grey bars) contents of *P. padus* leaves were measured in darkness so that the measuring heads of Dualex were either kept loosely together or pressed strongly together. The averages (n=6) are significantly different with 99.9 % (***) or 95 % (*) probability, calculated with Student's t-test. The error bars show SD calculated from 3‒6 technical repetitions and they are shown when larger than the symbols.
